# Microelectrode Array based Functional Testing of Pancreatic Islet Cells

**DOI:** 10.3390/mi11050507

**Published:** 2020-05-17

**Authors:** Ahmad Alassaf, Matthew Ishahak, Annie Bowles, Ashutosh Agarwal

**Affiliations:** 1 Department of Biomedical Engineering, University of Miami, Coral Gables, FL 33146, USA; a.alassaf@umiami.edu (A.A.); m.ishahak@umiami.edu (M.I.); abowles6@gatech.edu (A.B.); 2 DJTMF Biomedical Nanotechnology Institute, University of Miami, Miami, FL 33136, USA; 3 Department of Medical Equipment Technology, Majmaah University, Al Majmaah 11952, Saudi Arabia

**Keywords:** islets of Langerhans, insulin secretion, microelectrode array (MEA), glucose stimulated insulin response, electrochemical transduction

## Abstract

Electrophysiological techniques to characterize the functionality of islets of Langerhans have been limited to short-term, one-time recordings such as a patch clamp recording. We describe the use of microelectrode arrays (MEAs) to better understand the electrophysiology of dissociated islet cells in response to glucose in a real-time, non-invasive method over prolonged culture periods. Human islets were dissociated into singular cells and seeded onto MEA, which were cultured for up to 7 days. Immunofluorescent imaging revealed that several cellular subtypes of islets; β, δ, and γ cells were present after dissociation. At days 1, 3, 5, and 7 of culture, MEA recordings captured higher electrical activities of islet cells under 16.7 mM glucose (high glucose) than 1.1 mM glucose (low glucose) conditions. The fraction of the plateau phase (FOPP), which is the fraction of time with spiking activity recorded using the MEA, consistently showed distinguishably greater percentages of spiking activity with high glucose compared to the low glucose for all culture days. In parallel, glucose stimulated insulin secretion was measured revealing a diminished insulin response after day 3 of culture. Additionally, MEA spiking profiles were similar to the time course of insulin response when glucose concentration is switched from 1.1 to 16.7 mM. Our analyses suggest that extracellular recordings of dissociated islet cells using MEA is an effective approach to rapidly assess islet functionality, and could supplement standard assays such as glucose stimulate insulin response.

## 1. Introduction

Islets of Langerhans are three-dimensional (3D) multicellular clusters that range from 50 to 500 μm in diameter [1,2]. As the functional unit of the pancreas, islets maintain glucose homeostasis through the interdependent secretion of hormones from α, β, δ, and γ cells [3]. Dysfunction of the insulin-secreting β cells, arising from autoimmune destruction or insulin-resistance, results in the development of diabetes mellitus [4]. To elucidate the pathophysiology of diabetes, new approaches are being employed to study islets at the cellular level.

The electrophysiology of β cells has been investigated using a patch-clamp technique [5,6,7]. However, the disadvantages to performing the patch clamp technique are the invasiveness to the sample, technical complexity, and limited recording time (hours) [8]. Microelectrode arrays (MEAs), on the other hand, have been employed to collect electrical activities of islets [9,10,11,12,13]. The advantages of MEAs are the non-invasiveness to the sample, ease of execution, and higher duration of recording time (days) [13,14]. Pfeiffer et al. performed extracellular recordings of whole islets using MEA, a glass holding pipette angled at 30°, and a micromanipulator to control the islet location on top of the recording electrode. They were able to show continuous bursts of spikes at high glucose concentration (15 mM) and concluded that the length of the bursts correlated with the amount of insulin released. The fraction of a plateau phase (FOPP), which is the fraction of time with spiking activity recorded using the MEAs was developed as a metric for beta-cell function [9]. Schonecker et al. and Brouwer et al. also used comparable methods on whole islets to confirm that extracellular recordings correlated with intracellular electrical recordings. Phelps et al. reported a new method for culturing dissociated islet cells on glass coverslips, where they were able to perform detailed imaging studies by super-resolution and live cell microscopy. More importantly, cells in the dissociated human and rat islet cell monolayers (α, β, δ, and γ) were in proportions similar to native 3D islets [15].

Herein, we report MEA recordings of dissociated islet cells as an innovative method to capture the islet function while circumventing the limitations of the previously used techniques. Moreover, standard functional tests, i.e., glucose stimulated insulin secretion (GSIS) assays, were performed concomitantly. Our data correlated measurable parameters of electrical activities by the MEA to the functional secretory response of islet cells at the early time points of culture. Moreover, we were able to determine that detection of electrical activities of the islet cells in response to the high glucose stimulation were sustained throughout the long-term culture whereas insulin responses from GSIS were only detectable at the early time points. Together, this evidence supports the utility of MEA for measuring islet function in a highly sensitive, non-invasive, and real-time manner.

## 2. Materials and Methods 

### 2.1. Islets Dissociation and Culture

Human pancreatic islets were procured from organ donors at the Human Islet Cell Processing Facility at the Diabetes Research Institute (University of Miami, Miller School of Medicine, Miami, FL, USA), under Institutional Review Board (IRB) approval for use of human tissue for research. Human islets are from approved cadaveric organ donors from which at least one other organ has been approved for transplantation. Since the donors are brain dead, the IRB’s from the institutions that isolate the islets consider the tissue as “Exempt” from Human Studies Approval. In this study, islets were obtained from two normal non-diabetic donors, a 51 year old male and a 44 year old female, with body mass indices of 29.5 and 32.8 kg/m^2^, respectively.

Human collagen IV stock solution (1 mg/mL, Sigma Aldrich, St. Louis, MO, USA) was prepared and diluted to 50 μg/mL into Hanks’ Balanced Salt Solution (HBSS) with Ca^2+^/Mg^2+^ (Life Technologies, Carlsbad, CA, USA). Prior to collagen coating, all MEAs were placed inside a UV ozone cleaner (Jelight) for 8 min in order to sterilize and activate the MEA surface for protein coating. A 100 μL drop of the diluted collagen was then added to each of the UV ozoned MEAs. MEAs were then incubated with collagen IV overnight in 37 °C and washed 3 times with HBSS with Ca^2+^/Mg^2+^ right before cells seeding.

For dissociation, islets were collected in a conical tube and centrifuged for 2 min at 800 rpm. 400 μL of warmed 0.05% trypsin (Gibco, Waltham, MA, USA) was used for dissociation after three washes with phosphate buffer saline (PBS) were completed. While islets were suspended in trypsin in a cryogenic vial, gentle agitation was applied to the vial in a 37 °C beads bath for 3 min to help with dissociation. After trypsinization for 3 min, 15 mL of neuronal medium was added to deactivate the trypsin and centrifuged for 6 min at 1400 rpm. Approximately 200 cells/mm^2^ were added to each collagen IV coated MEA.

Neuronal culture medium was prepared by supplementing minimum essential medium (MEM, Life Technologies, Carlsbad, CA, USA) with 5% fetal bovine serum (FBS), 2% B-27 (50×, Life Technologies), 1% Penicillin-Streptomycin (100×, Life Technologies), 1% HEPES (1 M, Life Technologies), 1% Glutamax (100×, Life Technologies), 1% Na-pyruvate (100 mM, Life Technologies), and final glucose (Life Technologies) concentration of either 1.1 mM glucose (low glucose media) or 16.7 mM glucose (high glucose media).

### 2.2. Glucose-Stimulated Insulin Secretion (GSIS) Assay

Insulin secretion was assessed by GSIS of dissociated islets under static incubation. Briefly, dissociated islets cultured on each MEA were incubated for one hour at 37 °C in low glucose media (1.1 mM) followed by a one hour incubation in high glucose media (16.7 mM). After each incubation period, a 500 μL sample of media was collected and insulin concentrations were measured using a human insulin enzyme-linked immunosorbent assay (ELISA) kit (Mercodia, Uppsala, Sweden) after diluting the samples 1:500 in deionized water to ensure measurements were within the range of the ELISA kit.

### 2.3. Electrophysiological Recordings

Electrical activity was recorded from the dissociated islets during GSIS assays using MEA2100 system (Multi Channel Systems MCS GmbH, Reutlingen, Germany). Dissociated islets were cultured on a standard microelectrode array chip (60MEA200/30IR–TI–GR, Multi Channel Systems) that fits inside the MEA2100 system, which was connected with a temperature (37 °C) controller and an interface board that linked the whole system to a PC computer (Multi Channel Systems). Recordings were done on days 1, 3, 5, and 7 post seeding for 15 min using low glucose media first and then high glucose media for each MEA chip. Multichannel experimenter and analyzer programs were used to do on-line recordings and off-line analysis of data, respectively. Electrical signal from each recording was filtered with a high pass filter (200 Hz) and then a low pass filter (4000 Hz), adapted from a previous study [13], and sampled at 25 kHz. A threshold of 10 times the standard deviation of the noise was set to determine a spike. We submit that we were, in fact, more stringent about the threshold compared to some studies that used 5–6 times the standard deviation of the average noise amplitude [13,16]. For spiking profile plots, a bin size of 10 s was used for the total 30 min of recording, where the total number of spikes in each of these 10 s windows was calculated (30 min recording = 180 windows of 10 s) and used to plot the spiking profile.

### 2.4. Immunofluorescent Staining

Dissociated islets from were cultured for 7 days on 18 mm glass coverslips (Electron Microscopy Sciences). On day 7, each coverslip was incubated for 10 min at room temperature with 4% ice cold paraformaldehyde (PFA) solution after a quick rinse with warm PBS. Three washes with PBS were followed, where each wash was for 5 min with gentle shaking. Next, 0.1% Triton X-100 with gentle shaking was applied for 10 min at room temperature to permeabilize the cells. Three washes with PBS were followed, where each wash was for 5 min with gentle shaking. Using PBS 10% and 1% donkey serum were prepared and used as a blocking buffer. Samples were incubated at room temperature in a dark place for 1 hour after a 200 µL drop of 10% blocking buffer was applied to each sample. Primary antibodies (rabbit anti-insulin antibody, rat anti-somatostatin antibody, and goat anti-pancreatic polypeptide antibody, all purchased from Abcam) solution was prepared as 1:200 dilution with 1% donkey serum and 0.25% Triton-X100. After incubation for 1 hour with the blocking buffer, 200 µL drop of the primary antibodies solution was applied to each sample and then placed inside dark 4 °C fridge overnight.

On the second day, three washes with PBS and 0.01% Triton-X100 were followed, where each wash was for 5 min with gentle shaking. Secondary antibodies (Alexa Fluor 488 donkey anti-rabbit (Life Technologies), Alexa Fluor 555 donkey anti-rat (Abcam), and Alexa Fluor 594 donkey anti-goat (Abcam) solution was prepared as 1:500 dilution in addition to 4′,6-diamidino-2-phenylindole (DAPI, 1:200), where all were mixed with 1% donkey serum and 0.25% Triton-X100. 200 µL drop of the secondary antibodies solution was applied to each sample and incubated for 1 hour at room temperature in a dark place. Coverslips were then rinsed with PBS three times and mounted onto glass slides with ProLong Gold Anti-Fade Reagent (Life Technologies) and sealed with nail polish after curing of the mountant. Stained dissociated islets were imaged on a Nikon Eclipse Ti inverted fluorescent microscope with an Andor Zyla sCMOS camera using a 60× oil immersion objective.

### 2.5. Statistical Analyses

All statistical analyses were performed on Prism v8 software (GraphPad, San Diego, CA, USA). Paired student *t*-tests were used for statistical comparisons between the low and high glucose conditions for the different days. All values were reported as the mean ± standard error of the mean unless reported otherwise, and *p* < 0.05 was considered statistically significant.

## 3. Results

### 3.1. Dissociation and Culture of Islets

**Figure 1:**
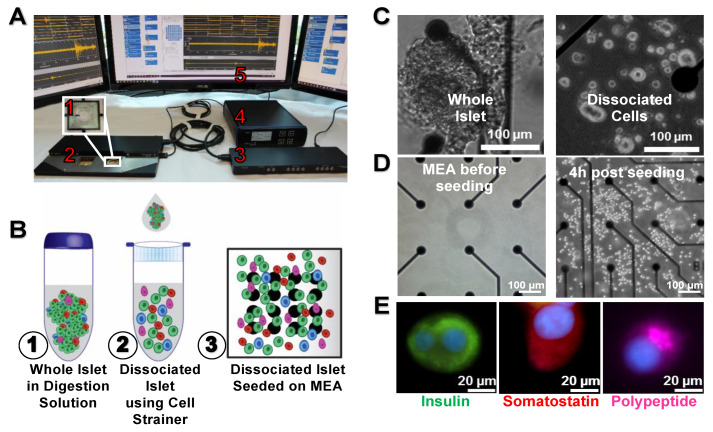
Dissociated islets on a microelectrode array (MEA). (**A**) MEA system setup contains five components; an MEA chip (A1), a two head-stages of MEA2100 system (A2), an interface board (A3), a temperature controller (A4), and a computer (A5). (**B**) Schematic illustration of clusters of whole islets in digestion solution (B1) dissociated into separated islets using a 40 µm cell strainer (B2), and finally seeding these dissociated islets on MEA coated with collagen IV (B3). (**C**) Bright field images of whole islet and dissociated islets on MEA. (**D**) Bright field images of MEA before seeding, and after four hours of seeding the dissociated islets. (**E**) Fluorescent images on day 7 for dissociated islets on glass coverslip showing successful separation of different cell population within the islet. Green color represents insulin (indicating β-cells), red color represents somatostatin (indicating δ-cells), magenta color represents pancreatic polypeptide (indicating γ-cells), and blue color represents DNA (indicating the cell nucleus).

Extracellular recordings of intact islets using MEA platform (Figure 1A) and classical MEA chips require proper contact and adhesion between cells and electrodes. Given that islets are large multicellular spheroids, limited contact area with the recording electrodes of planar MEA precludes MEA recording for functional evaluation of islets. Thus, we first enzymatically dissociated islets into single cells (Figure 1B,C) and cultured as adherent cells (hours; Figure 1D) on MEA electrodes to improve cell contacts and accuracy of recorded electrical activity. Fluorescence images on day 7 post seeding on the substrate (Figure 1E) showed that this technique dissociated and retained islet cell types, e.g., β, δ, and γ cells. After dissociation, extracellular recordings were performed the following day (day 1) and until the end culture (day 7).

### 3.2. Extracellular Recordings of Dissociated Islets

**Figure 2:**
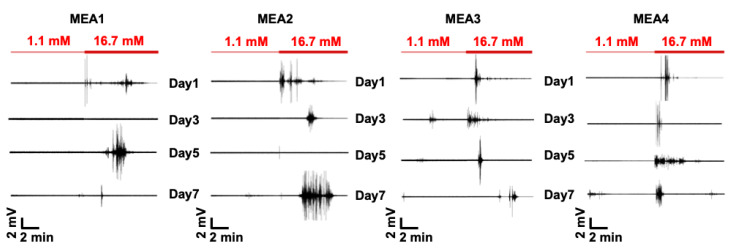
Electrical activity of dissociated human islets. Representative MEA recordings showing electrical activity of islet cells induced by switching from 1.1 to 16.7 mM glucose for different batches of human islets across 7 days.

**Figure 3:**
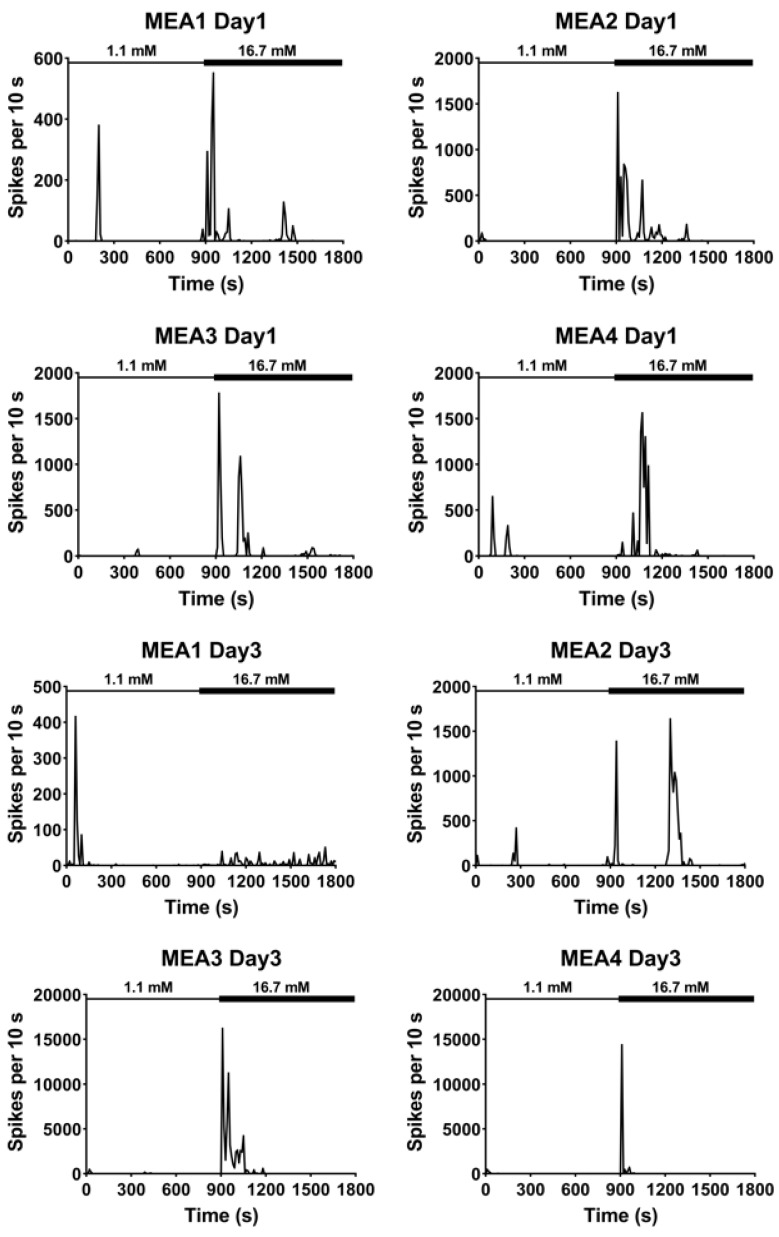

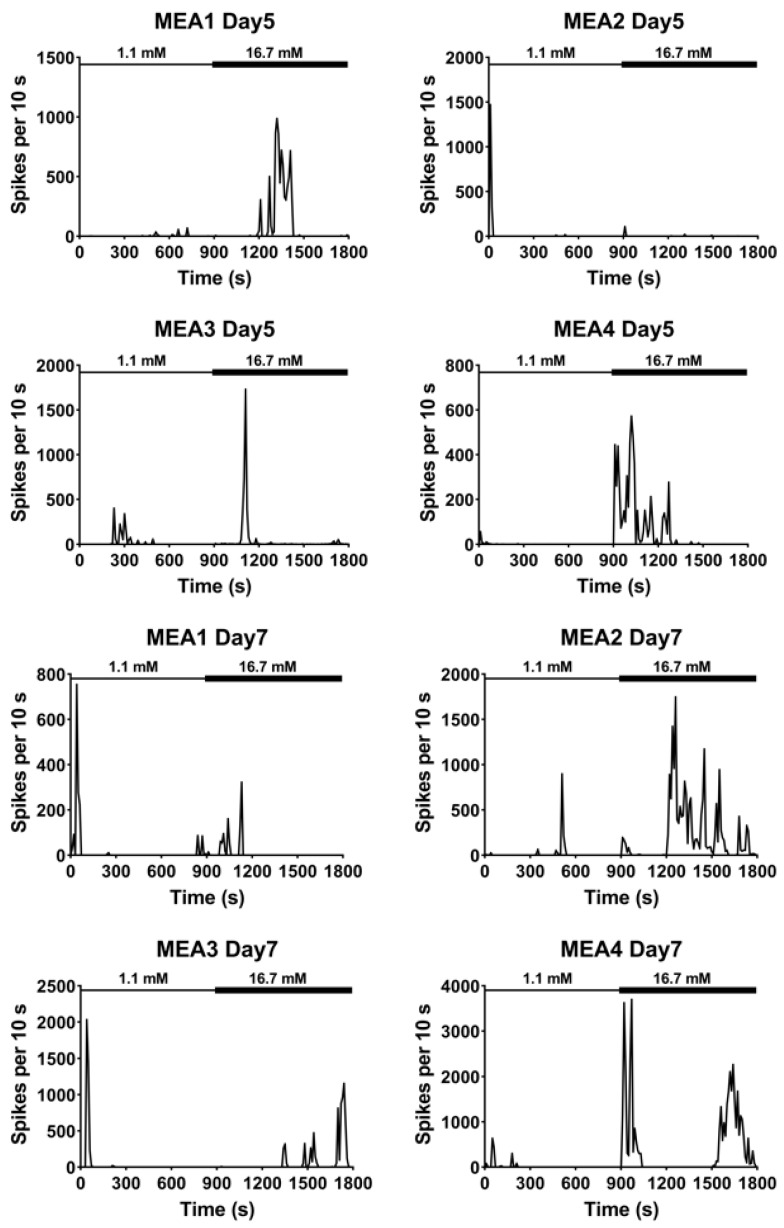
Total spiking with a bin size of 10 s for a total of 30 min recording. Each MEA was recorded with low glucose (1.1 mM) media for 900 s followed by another 900 s recording with high glucose (16.7 mM) media starting from day 1 post seeding until day 7. The number of spikes were summed every 10 s until the end of the recording.

Pancreatic β cells show oscillatory electrical activity known as slow waves in response to glucose [5,17]. Extracellular recordings using MEA were previously performed and compared to intracellular measurements obtained by traditional techniques with intact islets to demonstrate an alternate detection method to interrogate the electrical activity of islets [9]. MEA recordings performed using the dissociated islet cells that were seeded on four separate MEA chips (MEA1, MEA2, MEA3, and MEA4). The recordings were for a duration of 30 min on subsequent days, and they showed that high glucose induced longer electrical activity (minutes) with higher amplitudes (mV) compared to the low glucose recordings, which showed shorter electrical activity (seconds) with lower amplitudes (μV; Figure 2). MEA1 on day 3 and MEA2 on day 5, however, showed almost no electrical activity when the high glucose was introduced to the culture. Furthermore, the total number of spikes of the 30-minute recording from each MEA was quantified and the spiking profile plot was generated after binning the number of spikes every 10 s (Figure 3). Interestingly, the time course of binned spike profiles reveal electrophysiological activity that might be associated with the first (immediate) and second (sustained plateau) phase of insulin secretion that is usually seen during dynamic GSIS measurements [18].

### 3.3. Insulin Secretion and FOPP

**Figure 4:**
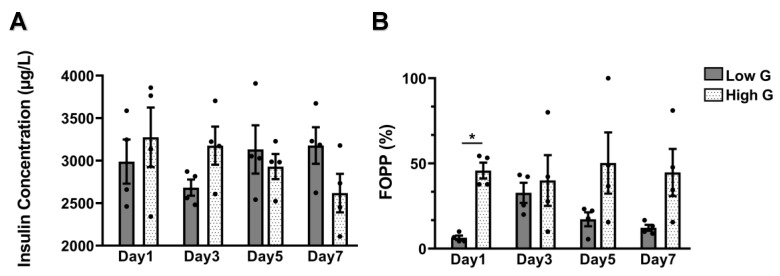
Assessment of insulin secretion and fraction of the plateau phase (FOPP). (**A**) Mean insulin concentration under static incubation of 4 released by dissociated islets on MEAs in low (1.1 mM) and high (16.7 mM) glucose media for different culture days (*n* = 4 MEAs for each condition). (**B**) Quantification of the mean FOPP measured by MEAs with dissociated islets in response to low (1.1 mM) and high (16.7 mM) glucose media for the different culture days (*n* = 4 MEAs for each condition). *p < 0.05.

β cells release insulin in response to varying blood glucose levels in vivo, thus measuring insulin concentrations upon exposure to varying levels of glucose in vitro is used as a method to correlate islet function. Standard functional testing using GSIS assays were performed simultaneously to the MEA recordings. Measured insulin concentrations under low (2989 ± 260 μg/L) and high (3275 ± 350 μg/L) glucose at day 1 were comparable to low (2683 ± 95 μg/L) and high (3177 ± 225 μg/L) glucose at day 3 (Figure 4A). However, insulin secretion decreased by day 5 (low G: 3132 ± 284 μg/L, and high G: 2930 ± 147 μg/L) and day 7 (low G: 3178 ± 215 μg/L, and high G: 2618 ± 226 μg/L) of culture. Figure S1 shows the insulin concentrations measurements for each MEA. Interestingly, functional testing using MEA showed FOPP measurements with continuous and high spiking activity with high glucose relative to the low glucose for all culture days. The FOPP calculations were low G: 6% ± 1.3% and high G: 46% ± 4.7% for day 1, low G: 33% ± 5.9% and high G: 40% ± 14.9% for day 3, low G: 17% ± 4.0% and high G: 50% ± 18% for day 5, and low G: 12% ± 1.8% and high G: 44% ± 13.8% for day 7 (Figure 4B). Even though the significant difference between the high and low glucose groups was only on day 1, but the other days showed distinguishable difference. Figure S2 shows the FOPP calculations for each MEA separately.

## 4. Discussion

Diabetes mellitus is estimated to affect over 400 million people worldwide by 2030 making it one of the most common and costly chronic diseases [3]. Diabetes is characterized by hyperglycemia related to autoimmune destruction of insulin-secreting β cells (type 1) or insulin resistance (type 2) [19,20]. Islet transplantation is a therapeutic alternative for β-cell replacement, which restores glycemic control in type 1 diabetes patients [21,22]. Islet function can be investigated by traditional assay such as GSIS [18], or by utilizing emerging MEA technology [11,23], which provides information about the islet electrophysiology to test the islet function.

Elucidating the complex and dynamic physiologic processes of healthy islets is imperative prior to transplantation. In general, varying blood glucose levels lead to changes in the membrane potential of β cells inducing an electrochemical mechanism resulting in the release of insulin [12,24,25,26]. More specifically, increased blood glucose concentration fuels glucose metabolism within β cells, and the product of glycolysis is adenosine triphosphate (ATP). The produced ATP reduces the resting membrane potential, which leads to membrane depolarization (electrical activity). After membrane depolarization, the voltage-gated Ca^2+^ channels open, increasing intracellular Ca^2+^ concentrations, which trigger fusion of vesicles containing insulin with the cell membrane, and subsequent exocytosis of insulin. Insulin is then released into the blood to allow all cells of the body to utilize glucose for energy [5,12].

MEA recordings using human islets were previously measured and electrical activity was detected with high glucose concentration [23,27]. Schonecker et al. showed no electrical activity corresponded to 1 mM glucose, while oscillatory activity (60–80 μV) was evoked by 10 mM glucose concentration when recording from whole islets [23]. In our study, moderate electrical activity (μV) was seen with 1.1 mM glucose, while high spiking (mV) was observed under 16.7 mM glucose concentration. The difference in amplitude between our study and the Schonecker et al. study may be inferred by several factors. Firstly, the Schonecker et al. study used the whole islet, which means only a small portion of the islet surface was in contact with the electrode, whereas we used dissociated islet that had more cells surface area in contact with an electrode. While our seeding density was 50,000 cells on the MEA, the Schonecker et al. study recorded from one whole islet. Additionally, where the Schonecker et al study used only a low pass filter of 100 Hz to filter their signal, compared to our study that used a high pass 200 Hz filter followed by a low pass 4000 Hz filter, which was adapted from Raoux et al’ study. Lastly, Schonecker et al. used mouse islets, while our studies exclusively utilized human islet cells.

Insulin exocytosis has been studied and known to follow a biphasic time course [24,28,29,30,31]. The first phase linked to a rapid transient increase rate of insulin secretion, commonly within 5 min of glucose stimulation. Then, insulin secretion decreased to a plateau (second phase) before it completely stopped with the end of glucose stimulus. This data suggests that the spiking initiation of this first and second phases in some of the MEAs from the extracellular recording when we plotted the total 30 min spiking profile after we used a 10 s bin size of and calculated the total number of spikes in each MEA. This is the first study to date that correlates spiking profiles to islet function during glucose stimulated insulin secretion of dissociated islets demonstrating an attractive use of MEA.

The length of the spiking when using MEA has been correlated with the amount of insulin released during glucose stimulation [9,12]. Therefore, FOPP assessment for all MEAs on the different days always showed higher percentages of FOPP during the high glucose condition compared to the low glucose. This evidence supports using MEA as a highly sensitive and robust tool to measure the function of human islets. Insulin secretion measurements using the conventional GSIS assay, on the other hand, showed the same trend between high glucose and low glucose conditions only on the early time points of culture. This could suggest the need for cell–cell contact, which was lost in the dissociated islets, and the cooperating cells may require direct intercommunication to secrete higher levels of insulin under high glucose concentrations. Together, these spiking profiles that correspond to glucose stimulated insulin secretion validates the use of MEA for examining islet function.

This study provided supportive evidence that extracellular recordings using MEA is non-invasive and a quick approach that could be used to test islet functionality. By dissociating the islets, the individual islet cells can be cultured, monitored, and recorded for an extended period, which was not possible before MEA technology. Future improvement to this platform includes seeding at various densities to determine the correlation with spiking activity and physiological insulin levels from islets cells. The next logical step to this study and previous MEA studies should be recording from MEA-based in vitro disease models (diabetic islets) and comparing that with the normal healthy islets (baseline) was done in this study. Diabetic islets once obtained, the use of diazoxide, tolbutamide, and KCL could be investigated to show their effect on the electrical activity. Furthermore, our methods can be used to screen new drugs as well as evaluate some of intervention strategies that could be performed when islets are chronically challenged by glucolipotoxicity or stress-inducing agents.

## 5. Conclusions

In conclusion, we demonstrated that extracellular recordings of dissociated islet cells using MEA is an effective approach to rapidly assess islet functionality, and could supplement standard assays such as glucose stimulate insulin response. Evidence from this study demonstrated islet dissociation, and creating layer of islet cells on planar MEA electrodes is critical for the assay. MEA recordings showed more electrical activity and FOPP percentages induced by the high glucose compared to the low glucose recordings. Furthermore, spiking profile plots from multiple MEA recordings revealed the electrophysiological activity that precedes the initiation of the first (immediate) phase and second (sustained) phase of insulin secretion usually seen during dynamic GSIS measurements. Our approach of dissociating the islets and utilizing the MEA platform to non-invasively test islet functionality is enabling detailed efforts to study islet physiology and screen potential pharmacological interventions

## Supplementary Materials

The following are available online at https://www.mdpi.com/2072-666X/11/5/507/s1, Figure S1: Glucose stimulated insulin secretion for functional assessments. Insulin concentration by dissociated islets on each MEA under low (1.1 mM) and high (16.7 mM) glucose media for the different culture days, Figure S2: Assessment of FOPP. Quantification of FOPP of each MEA for low (1.1 mM) and high (16.7 mM) glucose media for the different culture days.
