# Supplementary material for: Microelectrode Array based Functional Testing of Pancreatic Islet Cells
Source: Micromachines (Basel). 2020 May 17;11(5):507. doi: 10.3390/mi11050507 (PMC7281363; doi:10.3390/mi11050507)
Supplement: Supplementary file 1 [file micromachines-11-00507-s001.pdf]

micromachines

Supplementary Information: Microelectrode Array based Functional Testing of Pancreatic Islet Cells

Ahmad Alassaf [1,2,3], Matthew Ishahak [1,2], Annie Bowles [1,2] and Ashutosh Agarwal [1,2,*]

[1] Department of Biomedical Engineering, University of Miami, Coral Gables, FL 33146, USA; a.alassaf@umiami.edu (A.A.); m.ishahak@umiami.edu (M.I.); abowles6@gatech.edu (A.B.)
[2] DJTMF Biomedical Nanotechnology Institute, University of Miami, Miami, FL 33136, USA
[3] Department of Medical Equipment Technology, Majmaah University, Al Majmaah 11952, Saudi Arabia
* Correspondence: A.agarwal2@miami.edu; Tel.: 305-243-8925

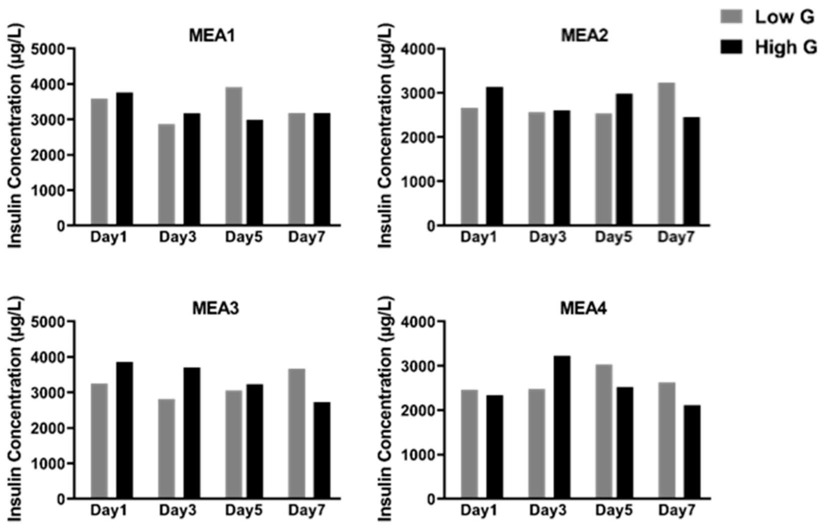

Figure S1. Glucose stimulated insulin secretion for functional assessments. Insulin concentration by dissociated islets on each MEA under low (1.1 mM) and high (16.7 mM) glucose media for the different culture days.

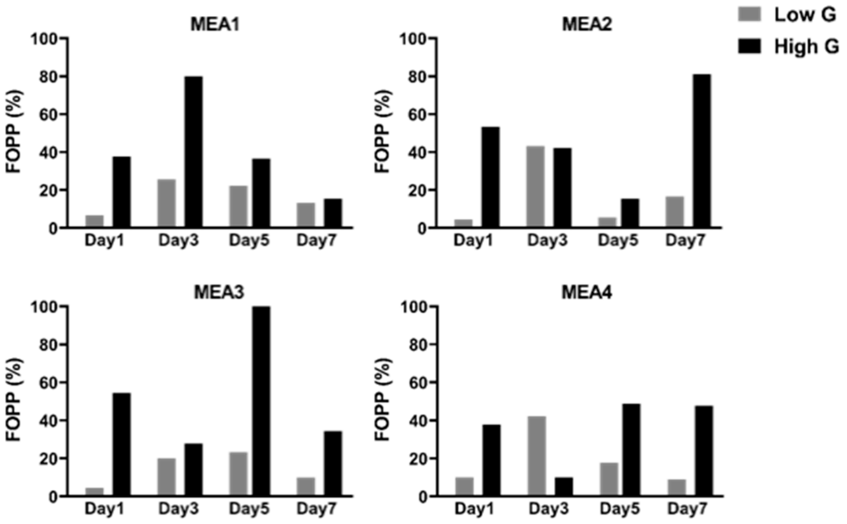

Figure S2. Assessment of FOPP. Quantification of FOPP of each MEA for low (1.1 mM) and high (16.7 mM) glucose media for the different culture days.
